# Prospective evaluation of artemether-lumefantrine for the treatment of non-falciparum and mixed-species malaria in Gabon

**DOI:** 10.1186/1475-2875-11-120

**Published:** 2012-07-10

**Authors:** Ghyslain Mombo-Ngoma, Christian Kleine, Arti Basra, Heike Würbel, Daisy A Diop, Mesküre Capan, Ayola A Adegnika, Florian Kurth, Benjamin Mordmüller, Fanny Joanny, Peter G Kremsner, Michael Ramharter, Sabine Bélard

**Affiliations:** 1 Medical Research Unit, Albert Schweitzer Hospital, Lambaréné, Gabon; 2 Institute of Tropical Medicine, University of Tübingen, Tübingen, Germany; 3 Département de Parasitologie-Mycologie, Faculté de Médecine, Université des Sciences de la Santé, Libreville, Gabon; 4 Department of Infectious Diseases, J.W. Goethe University Hospital, Frankfurt a. Main, Germany; 5 Department of Medicine I, Division of Infectious Diseases and Tropical Medicine, Medical University of Vienna, Vienna, Austria; 6 Department of Parasitology, Leiden University Medical Center, Leiden, The Netherlands; 7 Department of Infectious Diseases and Pulmonary Medicine, Charité-Universitätsmedizin Berlin, Berlin, Germany; 8 Centre for Paediatrics and Adolescent Medicine, University Medical Centre Freiburg, Freiburg, Germany

**Keywords:** Malaria, Ovale, Malariae, Artemisinin-combination-therapy, Artemether-lumefantrine, Non-falciparum

## Abstract

**Background:**

The recommendation of artemisinin combination therapy (ACT) as first-line treatment for uncomplicated falciparum malaria is supported by a plethora of high quality clinical trials. However, their recommendation for the treatment of mixed-species malaria and the large-scale use for the treatment of non-falciparum malaria in endemic regions is based on anecdotal rather than systematic clinical evidence.

**Methods:**

This study prospectively observed the efficacy of artemether-lumefantrine for the treatment of uncomplicated non-falciparum or mixed-species malaria in two routine district hospitals in the Central African country of Gabon.

**Results:**

Forty patients suffering from uncomplicated *Plasmodium malariae, Plasmodium ovale* or mixed-species malaria (including *Plasmodium falciparum*) presenting at the hospital received artemether-lumefantrine treatment and were followed up. All evaluable patients (n = 38) showed an adequate clinical and parasitological response on Day 28 after oral treatment with artemether-lumefantrine (95% confidence interval: 0.91,1). All adverse events were of mild to moderate intensity and completely resolved by the end of study.

**Conclusions:**

This first systematic assessment of artemether-lumefantrine treatment for *P. malariae*, *P. ovale* and mixed-species malaria demonstrated a high cure rate of 100% and a favourable tolerability profile, and thus lends support to the practice of treating non-falciparum or mixed-species malaria, or all cases of malaria without definite species differentiation, with artemether-lumefantrine in Gabon.

**Trial Registration:**

ClinicalTrials.gov Identifier: NCT00725777

## Background

Effective treatment of malaria is one of the main tools to control and eventually eradicate malaria. A plethora of clinical trials demonstrating high efficacy, satisfying effectiveness, and good tolerability and safety of artemisinin combination therapy (ACT) – the current first-line treatment of falciparum malaria – has been conducted and published over the past decade [1,2]. Treatment recommendations for non-falciparum malaria have however remained virtually unchanged since the introduction of chloroquine more than five decades ago [3].

*Plasmodium falciparum* and to a lesser extent *Plasmodium vivax* have attracted more scientific interest compared to the other human plasmodial species due to their high incidence, virulence and the emergence of drug resistant isolates. Today *Plasmodium ovale* and *Plasmodium malariae* are among the most neglected tropical diseases particularly in sub-Saharan Africa. This fact is illustrated by an analysis of clinical trials on *P. ovale* and *P. malariae* malaria resulting in less than five interventional clinical trials over the past 10 years, compared to 930 reports of clinical trials for *P. falciparum* malaria [4]. More research activities are, therefore, needed to boost the development of novel and improved control tools for these plasmodial infections.

Today chloroquine remains the standard of care for *P. malariae* and *P. ovale* malaria due to its low cost and sustained efficacy. ACT is recommended as first-line treatment for *P. falciparum* malaria and for mixed plasmodial infections [3]. Despite these official recommendations, ACT is used in many areas for the treatment of all forms of malaria including non-falciparum infections. This may be explained by the fact that non-falciparum species often occur concurrently with *P. falciparum* as mixed infections in sub-Saharan Africa [5-7], and that clinicians prefer the prescription of an ACT for malaria cases due to lacking or unreliable species differentiation. Finally, chloroquine treatment is unpopular in many parts of sub-Saharan Africa due frequently experienced adverse effects, e.g. induced pruritus.

At present, artemether-lumefantrine is the most widely used ACT worldwide with 130 million treatment courses procured in 2010 [8]. Anecdotal evidence indicates that artemether-lumefantrine may be highly efficacious against *P. malariaeP. ovale* and mixed-species malaria, however, a prospective evaluation is still lacking. Since artemether-lumefantrine is used on a large scale for these indications in sub-Saharan Africa we assessed the efficacy of artemether-lumefantrine in patients suffering from uncomplicated malaria due to non-falciparum species or mixed malaria species infections presenting at two district hospitals in the Central African country of Gabon.

## Methods

This prospective study was performed in Gabon at the Medical Research Unit of the Albert Schweitzer Hospital in Lambaréné [9] and the Regional Hospital of Fougamou. This Central African region is characterized by perennial transmission of *P. falciparum*[10] and co-endemicity of *P. ovale* and *P. malariae*[5]. Young children and pregnant women are at highest risk for malaria associated morbidity and mortality in this region [11-13]. *Plasmodium falciparum* isolates are highly resistant to chloroquine and sensitivity to antifolate anti-malarials has decreased [14-16], hence ACT has become the first-line treatment of malaria [17]. The study followed the principles of the Declaration of Helsinki (5th revision) and was approved by the regional ethics committee (CERIL). Written informed consent was obtained prior to inclusion in the study from adult participants or from a legal representative of children below 21 years of age, and assent was obtained where applicable.

Participants older than six months presenting with fever or with a history of fever during the previous four days and for whom malaria was confirmed by a thick blood smear were considered for inclusion. Thick and thin blood smears were performed and patients positive for *P. ovale*, *P. malariae* or a mixed infection with more than one plasmodial species (including *P. falciparum)* at a density of below 200,000/μl blood were considered eligible for enrolment.

Patients were excluded from this study when presenting with a clinical condition requiring hospitalization, haemoglobin < 7 g/dl, history of hypersensitivity to artemether-lumefantrine, prior intake of any anti-malarial medication, or current pregnancy or breastfeeding. Demographic data, medical history, physical examination, and axillary temperature were recorded at inclusion. Blood was obtained for haemoglobin measurement (HemoCue AB, Ängelholm, Sweden), malaria diagnostics, and storage on filter papers for molecular analysis. Axillary temperature and blood films were sequentially taken twice daily (at hours 0, 8, 24, 36, 48 and 60) during the treatment period. Follow up visits were scheduled for clinical examination, haemoglobin measurement, and malaria diagnostics on Days 7, 14 and 28 after the start of treatment.

Oral treatment with the standard six doses artemether-lumefantrine regimen (Coartem® and Coartem Dispersible®) was administered on three consecutive days. Artemether-lumefantrine was given as tablets or dispersible tablets under supervision with a small amount of liquid and patients were encouraged to consume simultaneously fatty food following current recommendations by the manufacturer.

Due to the well-known limitations of microscopic malaria species differentiation in routine laboratories in sub-Saharan Africa, PCR analysis based on the sequence of the small subunit ribosomal RNA (ssrRNA) gene and optional sequencing were performed for final plasmodial species classification. Capillary blood was collected on filter paper (Whatmann®) for subsequent PCR analysis. DNA was extracted with a commercial extraction kit (QIAamp DNA Blood Mini Kit, Qiagen) following manufacturer instructions and stored at −20°C for further analysis. Species-specific nested PCR for *P. falciparumP. malariaeP. ovale curtisi* and *P. ovale wallikeri* was carried out as previously described [18-21]. In samples not showing an amplicon for further species differentiation, a fragment of caseinolytic protease C gene (clpc) or cytochrome b gene was amplified by nested PCR [22] and subsequently sequenced on an ABI3100 Genetic Analyzer.

The primary objective was to assess the efficacy of artemether-lumefantrine for the treatment of non-falciparum and mixed plasmodial infections. The primary efficacy endpoint was the adequate clinical and parasitological response on Day 28. Re-appearing parasitaemia was further classified by PCR genotyping as treatment failure or newly acquired infection [19] except for *P. ovale* infections where potential relapse from liver hypnozoites precludes such a distinction. Secondary endpoints included descriptive assessment of safety, tolerability, parasite clearance time (PCT) and fever clearance time (FCT).

A sample size of 40 patients was shown to be sufficient to demonstrate a lower 90% confidence interval of adequate clinical and parasitological response of at least 90% with a power of 0.8 assuming more than 98% efficacy on day 28. The intention to treat population (ITT) was defined as primary outcome for tolerability and safety analysis and comprised of all patients included in the study. Per protocol population (PP) was defined as primary efficacy outcome and consisted of all patients having received a full course of study medication who were not prematurely withdrawn from follow up.

Data were captured on paper case record forms and transcribed to an electronic database. All data were checked manually and further statistical analysis was performed using JMP 5.0 (SAS Institute Inc., NC, USA). Descriptive statistics of baseline characteristics and outcome measures were computed.

## Results

Between July 2008 and July 2010, 40 patients were included in this study. One patient decided to withdraw consent before treatment initiation and another participant was excluded from the final analysis because of reported concomitant chloroquine intake. All 39 patients completed 28 day follow up and final analysis was done for 38 patients (15 female participants, 39%). The age of patients ranged from two to 50 years, with eight patients aged less than five years and 19 between five and 12 years of age. Baseline parasitaemia ranged from 31 – 100,680 trophozoites per microliter capillary blood (median 777/μl) (Table 1). Based on microscopy thirty-two patients had mixed-species infections and 7 patients presented with a non-falciparum mono-infection.

**Table 1:** Patient characteristics

**Study flow**			
	Patients included	40	100%
	Evaluable patients	38	95%
**Baseline characteristics**			
	Females	15	39%
	Age^1^	8	5-14
	Children < 5 years of age	8	21%
	Systolic blood pressure (mmHg)^1^	100	90-110
	Heart rate (/min)^1^	88	68-104
	Haemoglobin (g/dl) ^1^	11.3	10.1-12.4
	Haemoglobin in children < 5 years (g/dl) ^1^	11.5	10.0-12.6
	Haemoglobin in patients > 5 years (g/dl) ^1^	11.3	10.2-12.6
	Asexual parasitaemia^1^	777	266-3053
	Asexual parasitaemia in children < 5 years^1^	480	278-742
	Asexual parasitaemia in patients > 5 years^1^	849	225-6400
	Participants presenting with fever	5	13%

Eight hours after initiation of anti-malarial treatment 12 (32%) patients had cleared parasites in peripheral blood. Further 19 patients (50%) showed parasite clearance at 24 hours and all patients (100%) had cleared parasites within 36 hours. Median parasite clearance time was 24 hours. Median parasite clearance time was comparable in children below 5 years of age and adults (Table 2). Patients with higher parasite densities on inclusion had longer parasite clearance times. Only a minority of patients presented with fever at inclusion (n = 5, 13%) and all cleared fever within 36 hours after initiation of treatment. All evaluable patients had an adequate clinical and parasitological response on Day 28 and the overall cure rate was therefore 100% (95% CI: 91-100%).

**Table 2:** Patient outcomes

**Outcome Parameters**			
	Patients with ACPR (n, 95% CI)	38	91-100%
	Parasite clearance time (hours) ^1^	24	8-24
	Parasite clearance time in children < 5 years (hours) ^1^	24	24-33
	Parasite clearance time in patients > 5 years (hours) ^1^	24	8-24
	Fever Clearance Time (n = 5, hours) ^1^	8	8-30
	Patients experiencing any serious adverse event	0	0%
	Patients experiencing any adverse event	8	20%
	Haemoglobin D28 (g/dl)^1^	11.6	10.6-12.3
	Haemoglobin D28 in children < 5 years (g/dl) ^1^	11.1	10.6-12.5
	Haemoglobin D28 in patients > 5 years (g/dl) ^1^	11.7	10.5-12.2

Tolerability and safety of the artemether-lumefantrine was assessed for all patients from inclusion to Day 28. No serious adverse event was recorded in the course of this clinical trial and eight patients experienced adverse events (diarrhoea, urinary tract infection, convulsion, vomiting, cephalgia, abdominal pain, fever, and lymphadenopathy). Five adverse events were of mild severity (urinary tract infection, vomiting, cephalgia, abdominal pain), the other three adverse events were of moderate severity, and all adverse events completely resolved by the end of follow-up. The only adverse event considered as possibly study drug related was the occurrence of one episode of vomiting on day 1 after first drug administration.

Capillary blood was obtained and further tested by PCR for molecular species differentiation. In this analysis 19 out of 39 samples were confirmed as non-falciparum or mixed species infection. In the remaining 20 samples PCR analysis demonstrated amplification of *P. falciparum* isolates only and no amplification of non-falciparum parasite-specific amplicons. Presence of *P. malariae* mono-infection and mixed species infection was demonstrated in one and 18 samples, respectively. Mixed infections were confirmed by PCR analysis indicating co-infection with *P. falciparum* and *P. ovale* (n = 12, 63%), *P. falciparum* and *P. malariae* (n = 3, 16%) and concurrent occurrence of all three species in three samples (n = 3, 16%). *P. ovale curtisi* (n = 9) as well as *P. ovale wallikeri* (n = 10) isolates were present in the samples, concurrent presence of *P. ovale curtisi* and *P. ovale wallikeri* was found in four patients.

## Discussion

This is the first prospective assessment of artemether-lumefantrine in the treatment of *P. ovale, P. malariae* and mixed-species infection with *P. falciparum*. The results demonstrate rapid parasite and fever clearance when assessed twice daily and a reassuringly high cure rate of 100% on Day 28. No clinical or parasitological treatment failure occurred during the 28 day follow up period. The primary hypothesis of an adequate clinical and parasitological response rate above 90% could, therefore, be confirmed. Although – at least in theory – the occurrence of recrudescent parasitaemia after Day 28 cannot be ruled out, a longer follow-up period was precluded due to the potential for relapse of *P. ovale* infections and associated problems in classification as treatment failure or reinfection in such circumstances. Anti-relapse therapy with primaquine in *P. ovale* infection is not commonly prescribed in Gabon due to the high risk for reinfection and safety concerns in G6PD deficient individuals.

A limitation of the study design may be seen in the lack of a control group. However, due to frequent occurrence of mixed infections including *P. falciparum,* a randomized controlled trial design comparing standard chloroquine treatment with artemether-lumefantrine was precluded due to concerns for patients’ safety. Future randomized clinical trials evaluating different forms of ACT versus chloroquine in the treatment of *P. malariae* or *P. ovale* mono-infections would however be highly desirable. Despite recent reports of delayed parasite clearance of *P. malariae* and *P. ovale* to chloroquine the curative efficacy of this drug appears to be maintained [23]. In analogy to mixed malaria infections of *P. falciparum* with *P. malariae* and *P. ovale* in Africa, co-endemicity of *P. vivax* and *P. falciparum* in South East Asia requires more rigorous assessment of the use of ACT in general, and artemether-lumefantrine in particular, for the treatment of *P. vivax* malaria [24,25]. Solid evidence of the efficacy of ACT in the treatment of non-falciparum malaria is essential before removal of chloroquine from the market as a cheap over the counter drug taken for undiagnosed and undifferentiated malaria cases.

The discrepancy between microscopic and molecular species classification as observed in this study is striking but not unexpected. Firstly, microscopic determination of mixed infections is prone to misclassification due to the relative predominance of *P. falciparum* trophozoites and often low parasitaemia of the non-falciparum species. Microscopic assessment may, therefore, under- or over- estimate mixed species infections. To minimize this source of misclassification internal and external quality control systems for microscopic malaria diagnosis are present in most African malaria research institutions, but not in smaller hospitals or other primary care facilities. In this study, microscopists were under external quality assessment and an expert senior microscopist was available to decide on discrepant results. However, despite these measures a significant proportion of patients showed discrepant results in microscopy and PCR. This discrepancy is likely to be even more important in routine laboratories without the additional quality control mechanisms. This fact further underlines potential advantages of a single treatment algorithm for all plasmodial species since misclassification may lead to the prescription of inadequate and potentially ineffective anti-malarials. Molecular determination of non-falciparum and mixed species infection may also lead to misclassification. The phenomenon of low sensitivity for the detection of mixed species and non-falciparum infections by PCR was previously described and may be explained by multiple factors [26]. DNA recovery of low-level parasitaemia in mixed infections may be too low, potentially leading to an under-estimation of mixed infections. Use of frozen blood samples instead of filter paper sampling may improve the yield of DNA extraction and should be compared to dried blood samples in future studies of mixed infections. Most importantly, no difference in the clinical or parasitological response was noticed for PCR confirmed and un-confirmed cases. Hence, artemether-lumefantrine is as an excellent option for the treatment of patients with non-falciparum and mixed species infection. Since the prevalence of chloroquine resistance in Gabon is almost 100% [27], chloroquine treatment of those patients with sub-microscopic *P. falciparum* and microscopically detected non-falciparum mixed infection (n = 6 (15%) in this study) is likely to fail.

This data support the current practice of using artemisinin-combination therapy for the treatment of non-falciparum and mixed plasmodium infections or indeed clinically suspected malaria without prior species diagnosis in Central Africa. Besides high efficacy, proven safety and tolerability of artemether-lumefantrine, the use of this combination may also reduce the risk of treatment failure due to misdiagnosis of plasmodial species. However, contrary to our findings, two recent reports of clinical failures of artemether-lumefantrine treatment of *P. falciparum* and *P. malariae* mixed infections challenge this assumption [28,29]. Whether these reports are the rare exception or whether late recurrence of *P. malariae* constitutes a significant problem needs further evaluation.

Non-falciparum infections and mixed plasmodial species infections constitute a common infectious disease entity in tropical regions. In the setting of this study these infections account for an estimated 5-10% of malaria cases indicating the importance of non-falciparum and mixed species infections. With the exception of the recently conducted clinical development program of pyronaridine-artesunate combination therapy for the treatment of vivax malaria [30], non-falciparum malaria has been utterly neglected in anti-malarial drug development until these days accounting for less than 1% of published anti-malarial trials [4]. The conduct of clinical trials providing more evidence on the appropriate treatment of such infections therefore seems mandatory.

## Conclusions

In conclusion, this report constitutes the first prospective assessment of artemether-lumefantrine in the treatment of *P. ovale, P. malariae,* and mixed-species malaria. The demonstrated high cure rate of 100% and the proven favourable tolerability profile of artemether-lumefantrine lend support to the approach of treating non-falciparum or mixed species malaria or indeed all cases of malaria without the possibility of species differentiation with artemether-lumefantrine combination therapy in the Central African country of Gabon.

## Competing interests

The authors declare that they have no competing interests.

## Authors contributions

GMN contributed to data collection, interpretation of results, production and revision of the manuscript. CK contributed to data collection, production and revision of the manuscript. AB contributed to data collection, production and revision of the manuscript. HW contributed to data collection, production and revision of the manuscript. DA contributed to data collection, production and revision of the manuscript. MC contributed to data collection, production and revision of the manuscript. AAA contributed to conception and design, interpretation of data and revision of manuscript. FK contributed to conception and design, interpretation of data and revision of manuscript. BM contributed to data collection, interpretation of results, production and revision of manuscript. FJ contributed to data collection, interpretation of results, production and revision of manuscript. PGK contributed to conception and design and revision of manuscript. MR contributed to conception and design, interpretation of results, production and revision of manuscript. SB contributed to conception and design, data collection, interpretation of results, production and revision of manuscript. All authors read and approved the final manuscript.
